# Comparison of Passive Leg Raising and Intravenous Phenylephrine as Prophylaxis in the Prevention of Hypotension After Spinal Anaesthesia in Elective Caesarean Section: A Randomized Controlled Trial

**DOI:** 10.7759/cureus.80311

**Published:** 2025-03-09

**Authors:** Ajay Kumar, Madhuri Kurdi, Harshitha H, Kaushik Theerth

**Affiliations:** 1 Anaesthesiology, Shri Atal Bihari Vajpayee Medical College and Research Institution, Bengaluru, IND; 2 Anaesthesiology, Karnataka Medical College and Research Institute, Hubballi, IND; 3 Anaesthesiology, Medical Trust Hospital, Ernakulum, IND

**Keywords:** lower segment caesarean section, passive leg raise, phenylephrine infusion, post spinal hypotension, pregnancy

## Abstract

Background and objectives

Post-spinal hypotension (PSH) is most commonly encountered in patients undergoing lower-segment caesarean section (LSCS). Passive leg raising (PLR) has been attempted in septic shock to predict fluid responsiveness. PLR is a novel and less-tried manoeuvre to prevent PSH in patients undergoing LSCS. This study aimed to compare the efficacy of PLR and prophylactic phenylephrine infusion in preventing PSH.

Methods

With the Ethical Committee's approval and patient consent, this randomized controlled trial included 180 parturients undergoing elective LSCS. The effects of PLR and phenylephrine infusion were compared. Parturients in the leg elevation group received leg raising approximately 30 cm from the horizontal position with a wooden block for three minutes. Parturients in the phenylephrine group received phenylephrine infusion at 100 µg/minute for three minutes following subarachnoid block. Those in the control group did not receive any intervention. Baseline systolic and diastolic blood pressure and heart rate were recorded every minute for three minutes and then every three minutes until the delivery of the foetus. Fetal appearance, pulse, grimace, activity and respiration (APGAR) score were noted at the first and fifth minute following birth. Any episodes of hypotension, hypertension or bradycardia were recorded.

Results

The incidence of hypotension was maximum in the control group (n = 23, or 38.3%), followed by the phenylephrine group (n = 4, or 6.7%) and least in the leg elevation group (n = 3, or 5.0%). The difference in the incidence rates was statistically significant (p < 0.05). The incidence rates of hypertension and bradycardia were not significant in any of the groups. The neonatal outcome was the same in all three groups.

Conclusion

PLR is a better alternative to prophylactic phenylephrine infusion in preventing PSH.

## Introduction

Spinal anaesthesia for parturients who undergo caesarean section avoids the risks of general anaesthesia and provides better post-operative pain relief, while also keeping the woman awake to see her baby just after birth. However, spinal anaesthesia is associated with hypotension and bradycardia, which may be deleterious to both the parturient and the baby. Post-spinal hypotension (PSH) is common after spinal anaesthesia in mothers undergoing lower-segment caesarean section (LSCS), with an incidence of 55%-90% [1]. Such a high incidence is observed despite the partial left lateral decubitus position to limit the aortocaval compression caused by the gravid uterus [2]. Maternal hypotension is due to peripheral vasodilation and decreased vascular resistance, whereas bradycardia is secondary to a relative parasympathetic dominance, increased baroreceptor activity, or induction of the Bezold-Jarisch reflex.

When maternal systolic blood pressure (SBP) falls between 70 and 80 mmHg for four minutes, some foetuses develop sustained bradycardia with abnormal fetal heart rate patterns [3]. Maternal SBP <100 mmHg for 10 to 15 minutes may lead to placental hypo-perfusion, fetal acidosis and bradycardia [4].

Various prophylactic methods of preventing and treating hypotension, including proper patient positioning (lateral decubitus position), vasopressor support and intravenous fluids (crystalloids and colloids), have been tried. These were able to reduce hypotension but not eliminate it [5]. Several studies have shown that intravenous/intramuscular phenylephrine at various doses prevents PSH during LSCS [6-9]. Many investigations have been performed to identify an effective technique for the prevention of PSH; however, to date, no method has been found to prevent it totally [10,11]. One study showed that using leg compression immediately after spinal reduces the incidence of PSH [12]. Another study concluded that leg elevation decreases the incidence of PSH in LSCS [13].

This study aimed to compare two interventions, passive leg raising (PLR) and prophylactic intravenous phenylephrine infusion, to prevent PSH during LSCS. The primary objectives of this study were to compare the incidence of PSH with three different interventions: PLR, prophylactic phenylephrine infusion and control supine wedged position. The incidence of PSH was defined as the percentage of parturients who experienced at least one episode of hypotension from administering the spinal block to delivering the foetus. This study further aimed to determine the efficacies of the two interventions, PLR and prophylactic phenylephrine infusion, in preventing PSH. The secondary objectives were to estimate the incidence of maternal bradycardia and hypertension, note the neonatal outcome with the two interventions and compare it with that of the control group.

## Materials and methods

This randomized controlled trial was conducted over a period of one year after obtaining approval from the Hubli Ethical Committee of Karnataka Institute of Medical Sciences, Hubballi, India (approval no. KIMS/PGS/SYN/447/2017-18; dated November 3 and 4, 2017). The study was registered in the Clinical Trials Registry of India (CTRI/2020/11/028946).

The study included 180 parturients of the American Society of Anesthesiologists (ASA) physical status II, belonging to the age group of 18-45 years, with a singleton pregnancy of gestational age >36 weeks, weighing 40 to 90 kg, undergoing elective LSCS under spinal anaesthesia. The baseline blood pressure readings were systolic of 100-150 mmHg and diastolic of 70-90 mmHg, with a heart rate of 60-100 beats per minute. The exclusion criteria were height (<140 cm or >180 cm), pregnancy-induced hypertension, pre-eclampsia, any systemic or endocrine disorder, multiple gestations, fetal/placental abnormalities, contraindications for spinal anaesthesia and hypersensitivity to the drugs used in the study. After explaining the study technique, written informed consent was obtained from the parturients to use the data for research purposes.

The incidence rates of hypotension in PLR and prophylactic phenylephrine infusion were considered to be 34.7% (n = 26) and 23% (n = 6), respectively, from studies conducted by Hasanin et al. [13] and Ngan Kee et al. [9]. Accordingly, the sample size was calculated to be 180, with 60 members in each group (considering dropouts), an alpha value of 0.05, a power of 80% and a confidence interval of 95%.

The parturients were randomly assigned equally to three groups according to a computer-generated list of numbers: Group LE (leg elevation), Group PE (phenylephrine) and Group C (control).

Subjects who fulfilled the inclusion criteria were evaluated a day prior to the surgery, and details of the patient, i.e., name, age, weight and height, were noted. The parturient was kept nil per oral as per standard guidelines, and tablet ranitidine 150 mg was given to her on the evening before the surgery as per our institutional protocol.

On the morning of the surgery, an 18-gauge (G) intravenous cannula was secured - one on each upper limb - with the parturient in the supine position. The crystalloid preloading of 10 mL/kg of Ringer’s lactate was started via one of these cannulas 30 minutes prior to induction. Injection ranitidine 150 mg was given intravenously.

Standard monitors were attached to the patient. The patient’s non-invasive blood pressure, peripheral oxygen saturation and electrocardiogram were continuously monitored. The baseline of all these parameters was recorded and documented every minute for three minutes, and then every three minutes until the baby’s delivery.

Under aseptic precautions and with the patient in the sitting position, the back was painted and draped. The L3-L4/L2-L3 space was identified, a 25G spinal needle (Quincke-cutting edge type) was inserted, and the sub-arachnoid space was confirmed based on the free and clear flow of cerebrospinal fluid. Injection bupivacaine (0.5% heavy), 2 mL, was injected as per our institutional practice. The parturient was made supine, and a wedge was placed under her right flank to have a pelvic tilt of approximately 10 cm.

**Figure 1:**
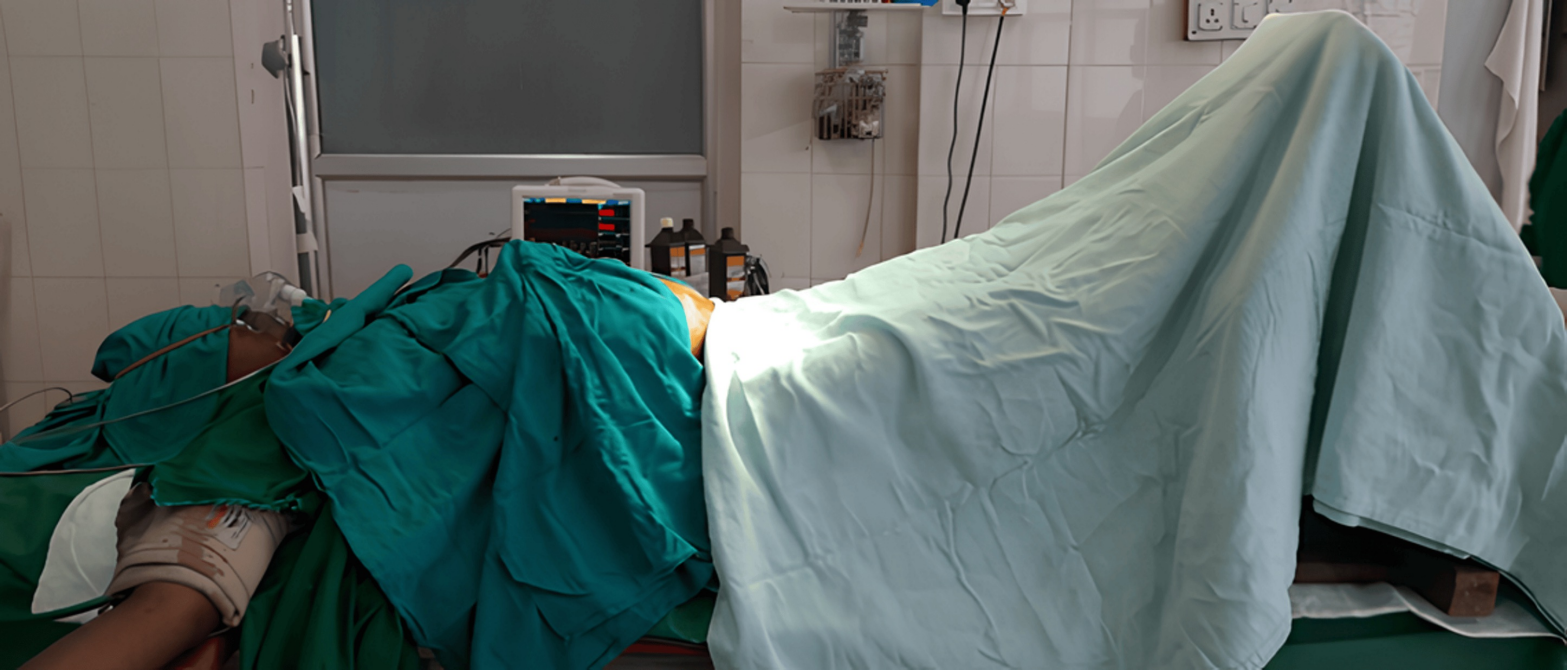
Leg elevation using wooden block done in this study

**Figure 2:**
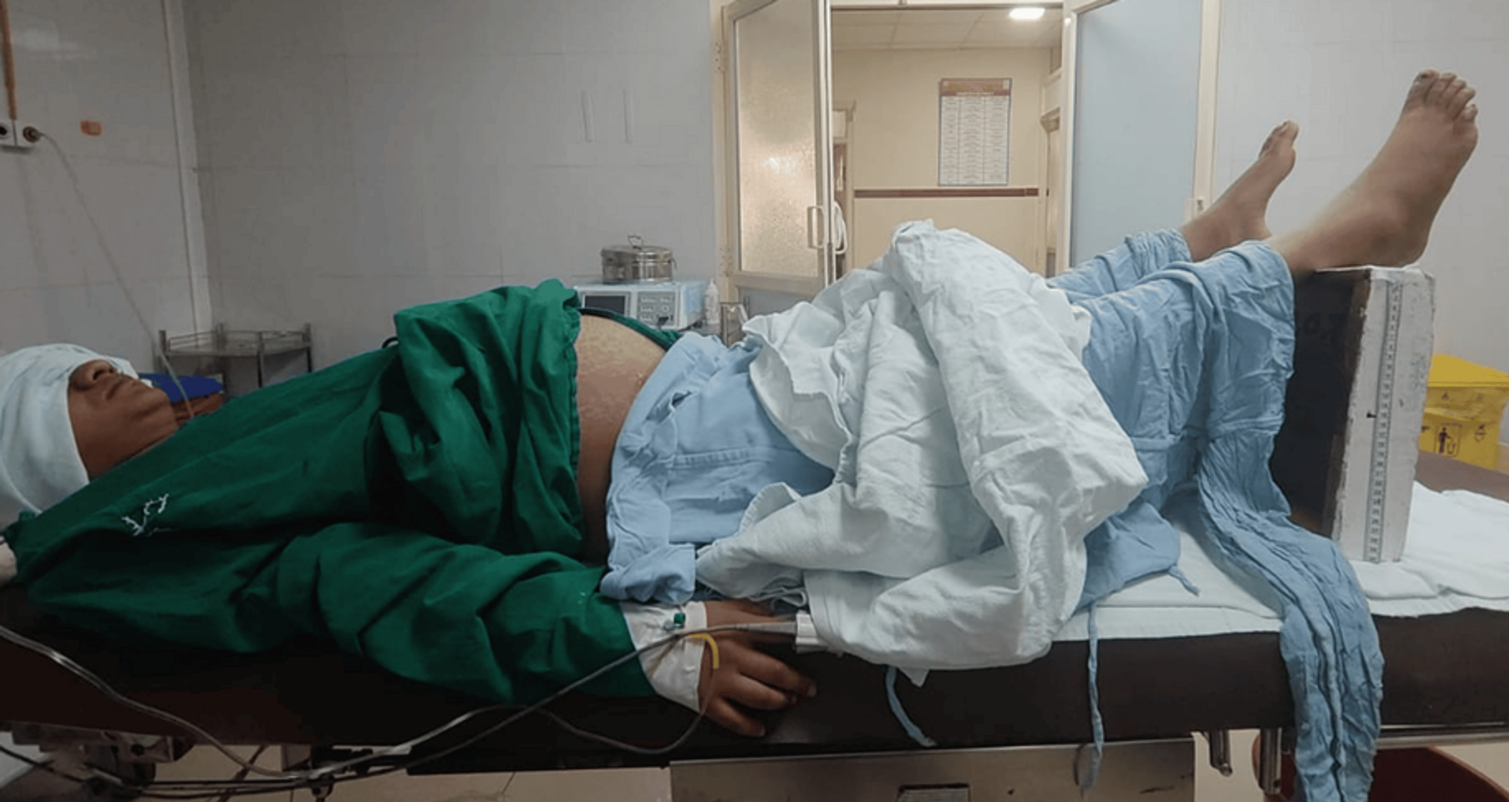
Leg elevation showing the wooden block applied in this study

**Figure 3:**
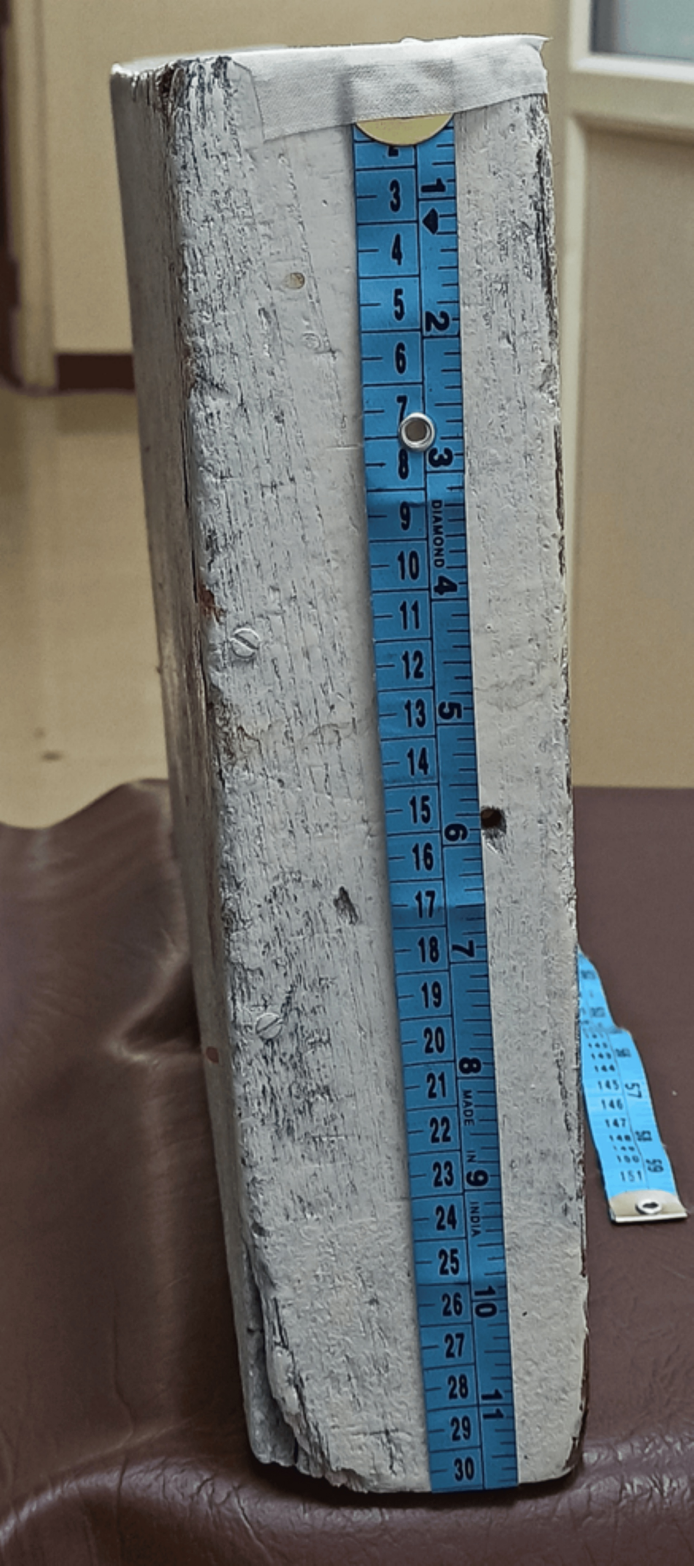
Wooden block of 30 cm height used in this study

Group LE patients received PLR of approximately 30 cm from horizontal with a wooden block for three minutes, and the block was removed promptly at the end of three minutes (Figures 1-3).

Group PE patients received a prophylactic phenylephrine infusion at the rate of 100 µg/minute via an infusion pump containing 10 mL of 100 µg/mL for three minutes via an 18G intravenous cannula. The infusion was stopped after three minutes. Group C patients remained in the supine wedged position throughout the surgery.

An oxygen face mask was applied, with an oxygen flow of 6 L/minute. Five minutes after the intrathecal injection, the upper level of the sensory block was assessed using the pin-prick method. By this time, the interventions were performed and stopped, the action level was checked, and the surgeon was allowed to paint and drape. The intrathecal injection, skin incision and delivery time were noted.

Episodes of hypotension, defined as SBP <90 mmHg or 25% from the baseline, were noted. The episode was treated with a 6 mg bolus dose of intravenous injection ephedrine, and the total dose required was recorded. Episodes of hypertension, characterized by blood pressure 20% greater than the baseline, were noted.

Episodes of bradycardia, defined as a heart rate of <50 beats per minute, were recorded. If an episode occurred in association with hypotension, it was treated with 0.6 mg intravenous atropine.

After delivering the foetus, intramuscular oxytocin 10 IU was administered as per institutional practice. The baby’s activity, pulse, grimace, appearance and respiration (APGAR) score at the first and fifth minute were recorded by the attending paediatrician.

Statistical analysis

IBM SPSS Statistics for Windows, Version 25 (Released 2017; IBM Corp., Armonk, NY, USA), was used to analyse the collected data. The analysis of variance test was used to compare the age, weight and height of the three groups. Changes in the blood pressure and heart rate within groups were analysed using ‘Bonferroni post-hoc analysis’, ‘Bonferroni correction’ or ‘Bonferroni adjustment’. The incidence rates of hypertension, hypotension, bradycardia and neonatal outcomes were compared using Fisher's exact test to determine the significance of the difference. A p-value of <0.05 was considered statistically significant.

## Results

**Figure 4:**
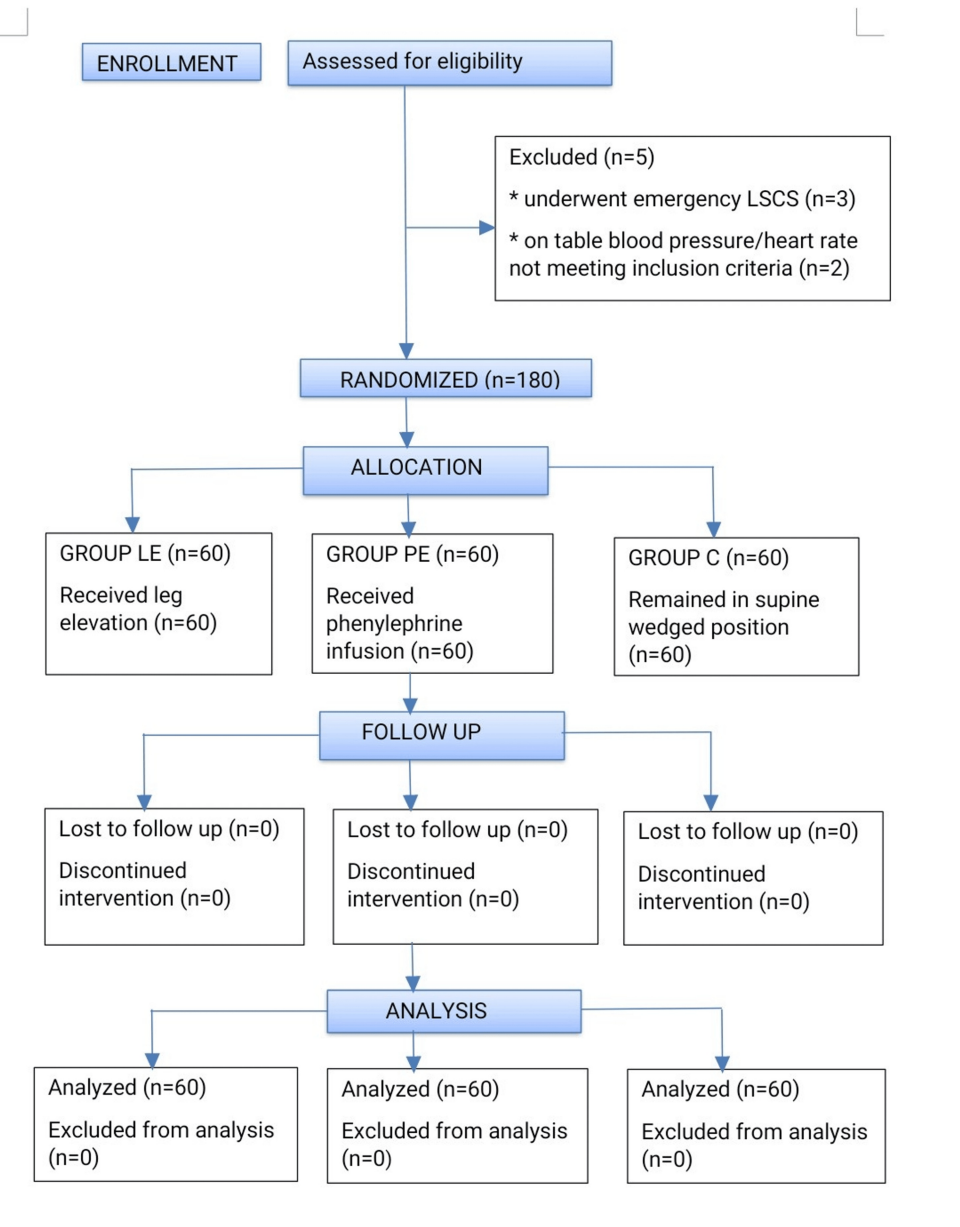
CONSORT diagram of the present study n: number of participants; LE: leg elevation group; PE: prophylactic phenylephrine group; C: control wedge group; LSCS: lower-segment caesarean section; CONSORT: consolidated standards of reporting trials

A total of 180 participants were enrolled in the study, with 60 each in Groups LE, PE and C (Figure 4). The demographic characteristics (age, weight, height and body mass index) (Table 1), ASA physical status class and the level of sensory block (T4-T6) following spinal anaesthesia were found to be comparable in the three groups (p > 0.05).

**Table 1:** Demographic characteristics of the study parturients Data expressed as mean (standard deviation) n: number of patients; LE: leg elevation; PE: phenylephrine infusion; C: control

Parameter	Group LE (n = 60)	Group PE (n = 60)	Group C (n = 60)	p-value
Age (years)	23.60 (2.99)	23.73 (3.17)	23.80 (2.91)	0.934
Weight (kg)	58.87 (6.35)	57.85 (4.99)	58.07 (5.20)	0.573
Height (cm)	156.50 (5.86)	157.68 (5.00)	157.57 (4.12)	0.369
BMI (kg/m^2^)	24.19 (3.60)	23.27 (1.78)	23.43 (2.37)	0.140

The intragroup analysis of mean systolic and diastolic blood pressure values showed a statistically significant (p < 0.05) decrease from baseline in all groups at the first and second minute (Tables 2-3). The decrease in diastolic blood pressure was seen till the 10th minute; however, there was no significant fall after the sixth minute.

**Table 2:** Comparison of systolic blood pressure values between the groups Baseline values were recorded on the table prior to administering spinal anaesthesia. Values were recorded at the first, second, third and sixth minute following spinal anaesthesia. NS: not significant (p > 0.05); HS: highly significant (p<0.001); SIG: significant (p < 0.05); LE: leg elevation; PE: phenylephrine; C: control

Interval	Groups	Systolic blood pressure		p-value	Significance
		Mean	Standard deviation		
Baseline	LE	116.40	8.76	0.611	NS
	PE	116.02	9.98		
	C	114.78	9.16		
1 minute	LE	105.13	10.09	0.000	HS
	PE	110.32	10.54		
	C	99.05	11.71		
2 minutes	LE	105.95	7.08	0.013	SIG
	PE	107.65	11.16		
	C	102.32	11.26		
3 minutes	LE	104.22	5.76	0.124	NS
	PE	106.02	11.33		
	C	107.77	10.35		
6 minutes	LE	104.05	6.55	0.711	NS
	PE	105.15	11.87		
	C	103.97	6.80		

**Table 3:** Comparison of diastolic blood pressure values between the groups Baseline values were recorded on the table prior to administering spinal anaesthesia. Values were recorded at the first, second, third and sixth minute following spinal anaesthesia. NS: not significant (p > 0.05); HS: highly significant (p<0.001); LE: leg elevation; PE: phenylephrine; C: control

Interval	Groups	Diastolic blood pressure		p-value	Significance
		Mean	Standard deviation		
Baseline	LE	83.43	9.92	0.059	NS
	PE	80.82	7.06		
	C	78.70	10.15		
1 minute	LE	74.78	9.68	0.000	HS
	PE	77.03	9.09		
	C	67.92	16.58		
2 minutes	LE	74.83	5.80	0.000	HS
	PE	74.85	8.30		
	C	66.83	13.61		
3 minutes	LE	71.35	5.57	0.160	NS
	PE	74.57	8.80		
	C	74.23	13.98		
6 minutes	LE	71.20	7.02	0.005	HS
	PE	71.12	8.22		
	C	66.98	8.50		

**Figure 5:**
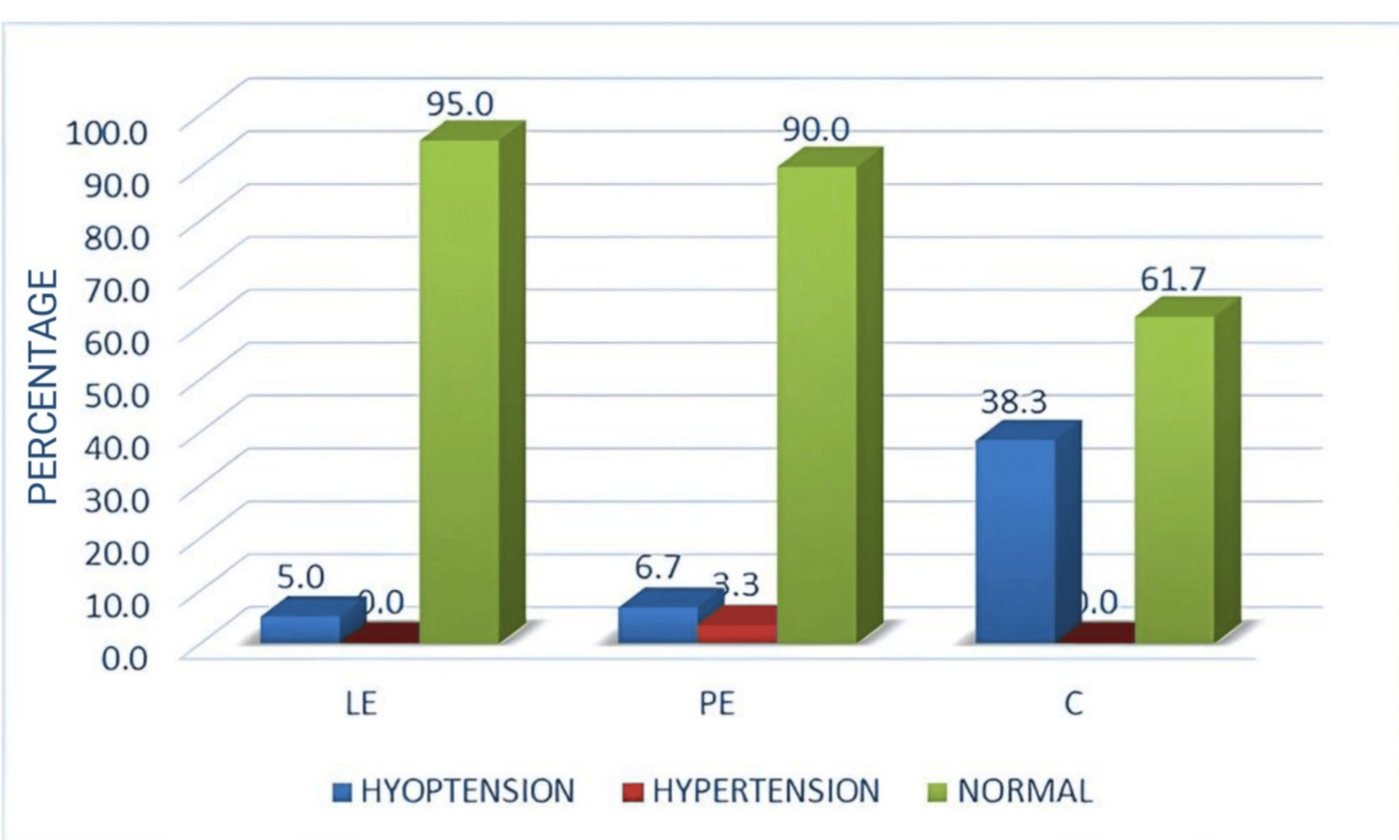
Incidence of hypotension and hypertension among the three groups LE: leg elevation; PE: phenylephrine infusion; C: control

The incidence of hypotension and requirement of ephedrine recorded for Group C were significantly higher (p < 0.05) than those for Groups PE and LE. Although Group LE exhibited the lowest incidence of hypotension, the mean hypotension value was lower in Group PE than in Group LE (Figure 5).

Although the occurrence of hypertension and bradycardia was seen only in Group PE, it was not statistically significant. The APGAR score at the fifth minute showed a statistical difference, with Group LE neonates having a better score. However, neonatal outcomes remained normal in all groups. No statistical differences in neonatal outcomes were observed among the three groups.

## Discussion

Spinal block results in the blockade of thoracolumbar sympathetic fibres, leading to profound vasodilation [14]. This decreases the arterial blood pressure and the venous return, consequently accentuating hypotension [15].

The treatment of PSH is one of the controversial topics in obstetric anaesthesia [16]. Several methods are available for preventing PSH in caesarean section. These include fluid preloading, lateral tilt, prophylactic vasopressors and leg elevation and compression. The major hemodynamic concern after spinal anaesthesia is inadequate venous return [17], and therefore, manoeuvres that increase it would help prevent PSH.

Vasopressors are used to treat hypotension after a subarachnoid block. Several researchers have studied various drugs for this purpose, including mephentermine, methoxamine, ephedrine, phenylephrine and norepinephrine.

This study primarily aimed to compare the effects of prophylactic PLR and prophylactic phenylephrine infusion in preventing PSH during caesarean delivery. The benefit of compression/raising of the legs following spinal injection is that it increases the venous return at the most required time and at a faster rate than can be achieved using intravenous infusion [18]. Leg elevation induces autotransfusion of blood from the lower extremities to the central circulation. Therefore, leg elevation increases the cardiac preload and hence, the cardiac output [19,20].

In 2017, Hasanin et al. [13] observed that leg elevation decreases the incidence of PSH in caesarean section. Theirs was a randomized controlled trial wherein 150 full-term parturients scheduled for caesarean section were randomized into two groups: Group LE and Group C. Group LE patients received leg elevation directly after spinal anaesthesia, which was maintained until skin incision. Intraoperative hemodynamic parameters, such as arterial blood pressure and heart rate, and intraoperative ephedrine consumption were recorded. Their study showed that Group LE exhibited a lower incidence of PSH than Group C. Ephedrine requirement in Group LE was lower than that in the non-elevation group.

More recent studies by Sari and Ozyurt [21] and Assen et al. [22] also found similar results, with the incidence of hypotension being lower in Group LE. Also, rescue ephedrine and phenylephrine use was lower with Group LE. This study also demonstrated that the incidence of hypotension was lower in Group LE (Figure 5) and that the ephedrine requirement was higher in Group C than in Groups PE and LE. However, a statistically significant difference in ephedrine consumption was not observed between Groups LE and PE. Importantly, although the incidence of hypotension was lower in Group LE in all the aforementioned studies, a marked difference in the incidence of hypotension was noted among the studies, with the incidence being only 5% (n = 3) in our study, 33.3% (n = 8) in Assen et al.'s study [22], 34.7% (n = 26) in Hasanin et al.'s study [13] and 41.4% (n = 29) in Sari and Ozyurt's study [21].

Our results differed from Rout et al.'s study [12], which found that patient leg elevation had no effect on the incidence of hypotension. Rout et al. included only 31 patients in each group, so their study could have been underpowered to prove this hypothesis. Our study has the advantage of a much larger study population of 60 patients undergoing passive leg elevation [12].

A study in 2019 by İnce et al. [23] showed similar results to ours. This study included 40 pregnant women aged 18-40 years scheduled for caesarean section under spinal anaesthesia. After spinal anaesthesia, patients in the PLR group were laid in the supine position, the operating table was set to a pre-determined position and the legs of the patients were raised to an angle of 30° with their waists. The patients in the control group were placed in the supine position. Patients’ hemodynamic values were recorded before and after spinal anaesthesia. Furthermore, the baby’s weight, APGAR score and blood gases were measured. Systolic arterial pressure was lower at four and six minutes in the control group and at 16 minutes in the PLR group. The number of patients requiring ephedrine, the total amount of ephedrine and the incidence of hypotension were significantly lower in the PLR group. These findings agree with those from the present study. Nonetheless, the legs of the patient were raised to 30 cm, viz 45° in our study cases, which was more than the elevation given to patients in İnce et al.’s study [23].

However, the degree and method of leg elevation tend to vary in different studies. Moreover, the duration for which the leg elevation has to be maintained remains unknown. In Hasanin et al.’s study [13], a leg elevation of 30 cm was performed using two standard pillows that were positioned under the heels, and the elevation was maintained until the skin incision. In their study, Rout et al. [12] performed leg elevation at an angle of 30° using four pillows until the completion of the surgery. In their study, İnce et al. [23] performed leg elevation with a pre-determined operating table of 30° initially and then lowered it to 15° during skin incision and maintained it throughout the surgery. Assen et al. [22] used two pillows to elevate the legs by 45° or 30 cm and maintained it until the end of the surgery. Sari and Ozyurt [21] used a cushion to raise the legs by 30°. In this study, a wooden block was used to achieve a leg elevation of 30 cm (Figure 1). Owing to the lack of consensus on the duration of leg elevation, it was maintained for three minutes considering the fact that obstetric spinal action usually occurs within three minutes. Also, the surgeons expressed concern about not being comfortable when performing the surgery on a patient in the leg elevation position. Incidentally, the onset and level of spinal action were tested in all our cases, and the onset was always within three minutes.

Recently, researchers have obtained promising results in preventing PSH by combining leg elevation with leg wrapping. Esen et al. [24] and Khedr [25] used Esmarch elastic bandages to perform leg wrapping immediately before spinal anaesthesia. Leg elevation was performed following intrathecal injection. Both studies concluded that combining wrapping and leg elevation improved the hemodynamics of the parturients.

Various studies have shown that alpha agonists such as phenylephrine are associated with better fetal acid-base status than ephedrine. Several studies have reported lower frequencies of patients with hypotension when using infusions of phenylephrine than when using those of ephedrine [26-30]. Hence, in our study, phenylephrine was selected as the vasopressor to be compared. Phenylephrine at 100 µg/minute was administered for three minutes to prevent PSH.

A randomized double-blinded controlled trial by Ngan Kee et al. [9] in 2004 showed that phenylephrine infusion at the rate of 100 µg/minute for three minutes immediately after intrathecal injection reduced the incidence of hypotension during spinal anaesthesia for caesarean delivery. Nonetheless, the incidence of hypotension was lower in Group PE in our study. However, the incidence of hypotension was higher when compared with Group LE.

A meta-analysis by Heesen et al. [7] concluded that phenylephrine alleviated the risk of hypotension, nausea and vomiting after spinal doses of bupivacaine >8 mg. However, there was no evidence that it reduced other maternal and neonatal morbidities. The incidence of hypotension was lower in the prophylactic phenylephrine group in our study patients, and they received hyperbaric bupivacaine 10 mg for spinal anaesthesia.

In the study by Hasanin et al. [13], the ephedrine requirement in the leg elevation group was lower than that in the non-elevation group. Nevertheless, the ephedrine requirement was higher in Group C than in Group PE and Group LE in our study. However, there was no statistically significant difference in ephedrine consumption between Group LE and Group PE.

Phenylephrine can cause severe bradycardia and hypertension in patients, which can be detrimental to the mother and the foetus. In our study, two patients developed bradycardia and hypertension, but they were successfully managed. Considering these adverse effects of phenylephrine, LE would definitely be a simple and effective manoeuvre for preventing PSH.

**Figure 6:**
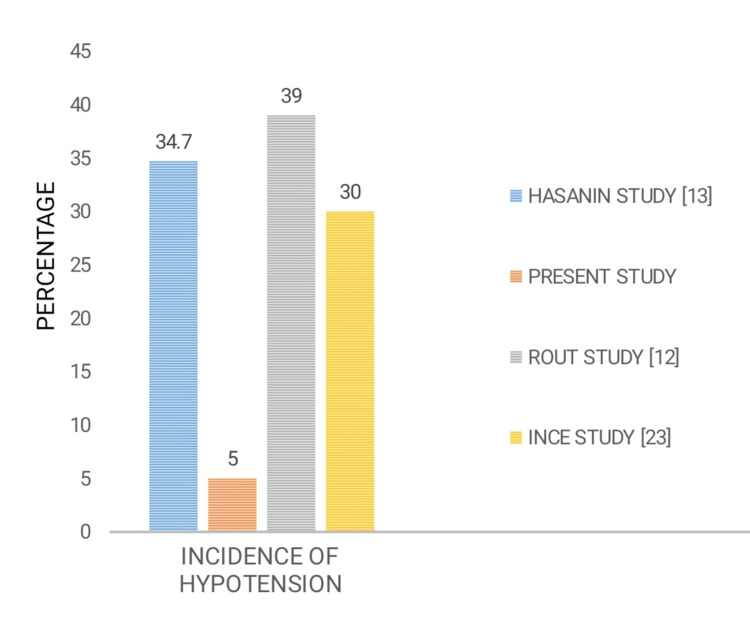
Incidence of hypotension in different studies Blue indicates Hasanin et al.'s study [13]; Orange indicates present study; Grey indicates Rout et al.'s study [12]; Yellow indicates İnce et al.'s study [23].

There is a dearth of studies comparing LE (physical intervention) and vasopressor infusions (pharmacological intervention) for preventing PSH in elective caesarean section. Our study compared both these techniques and found that the incidence of hypotension was lower in Group LE and that prophylactic leg elevation was better than prophylactic phenylephrine infusion (Figure 6). Using a wooden block to perform elevation helped maintain uniformity and provided a standard elevation angle, unlike other studies that used pillows and cushions which can get compressed with repeated use, the passage of time (studies maintaining elevation until the end of the surgery) and with the weight of lower limbs following spinal anaesthesia. In addition, leg elevation was provided in this study for three minutes, which has not been used by other researchers. We consider all these to be the strengths of our study.

Study limitations

Nonetheless, this study has certain limitations. Failing to standardize the injection rate of bupivacaine and inter-individual variability in the technique could have affected our findings. Increasing the frequency of blood pressure measurements and using advanced cardiac output monitors would have been more precise in studying the effect of leg elevation on maternal hemodynamics. These limitations, nonetheless, can be overcome in future studies.

## Conclusions

Regarding the prevention of PSH during elective LSCS, PLR is more efficacious than prophylactic phenylephrine infusion. The use of phenylephrine is associated with higher rates of maternal bradycardia and hypertension than PLR and the control supine wedged position. The fetal outcome, as assessed by the APGAR score, is similar in all three groups.
